# Aging with HIV vs. HIV Seroconversion at Older Age: A Diverse Population with Distinct Comorbidity Profiles

**DOI:** 10.1371/journal.pone.0118531

**Published:** 2015-04-13

**Authors:** Giovanni Guaraldi, Stefano Zona, Thomas D. Brothers, Federica Carli, Chiara Stentarelli, Giovanni Dolci, Antonella Santoro, Barbara Beghetto, Marianna Menozzi, Cristina Mussini, Julian Falutz

**Affiliations:** 1 University of Modena and Reggio Emilia, Modena, Italy; 2 Faculty of Medicine, Dalhousie University, Halifax, Canada; 3 McGill University Health Center, Montreal, Canada; Kliniken der Stadt Köln gGmbH, GERMANY

## Abstract

**Objective:**

People aging with HIV might have different health conditions compared with people who seroconverted at older ages. The study objective was to assess the prevalence of, and risk factors for, individual co-morbidities and multimorbidity (MM) between HIV-positive patients with a longer duration of HIV infection, and patients who seroconverted at an older age. We compared estimates across both groups to a matched community-based cohort sampled from the general population.

**Methods:**

We performed a case-control study including antiretroviral therapy (ART)–experienced patients who were HIV seropositive for ≥ 20.6 years (“HIV-Aging”), or who were seropositive for < 11.3 years (“HIV-Aged”) having access in 2013 at the Modena HIV Metabolic Clinic. Patients were matched in a 1:3 ratio with controls from the CINECA ARNO database. MM was defined as the concurrent presence of >2 NICM. Logistic regression models were constructed to evaluate associated predictors of NICM and MM.

**Results:**

We analysed 404 HIV-Aging and 404 HIV-Aged participants in comparison to 2424 controls. The mean age was 46.7±6.2 years, 28.9% were women. Prevalence of HIV co-morbidities and MM were significantly higher in the HIV-positive groups compared to the general population (p<0.001) and a trend towards higher rates of MM was found in aging vs aged group. This difference turned to be significant in patients above the age of 45 years old (p<0.001).

**Conclusions:**

People aging with HIV display heterogeneous health conditions. Host factors and duration of HIV infection are associated with increased risk of MM compared to the general population.

## INTRODUCTION

Better understanding of the intersection of HIV, aging and health is an urgent issue due to the increasing number of people aging with HIV[1, 2] as the synergistic result of two concurrent phenomenon: the increased life expectancy of people with HIV undergoing HAART, extensively demonstrated both in high[3, 4] and middle- and low-income countries[5, 6], but also the increasing number of people seroconverting HIV at an older age, as the result of a lower perception of sexual risk in older people[7, 8].

For instance, in Canada the number of older adults with HIV has doubled over the past 20 years and in the Western Europe the number of people living with HIV aged 50 years and over was estimated as almost quadrupled over the past decade[9]. Therefore the aging of HIV epidemic is a matter of fact, with potential concern because of physical, mental and psychological issues that can accompany both aging and HIV infection[10].

Aging with HIV is often linked to non-infectious comorbidities (NICM), including cardiovascular disease (CVD), hypertension, type 2 diabetes mellitus (T2DM), chronic kidney disease (CKD), osteopenia/osteoporosis, and non-AIDS cancers. These heterogeneous comorbidities share age and presumably HIV-infection as independent risk factors, and tend to aggregate into complex multi-morbidity patterns (usually defined as two or more NICM being present in the same individual concurrently)[11, 12].

Current literature focusing on HIV in older populations is concentrated among studies comparing older HIV-positive populations with younger HIV-positive individuals, or else comparing older HIV-positive individuals with older HIV-negative individuals[1, 13, 14]. Such comparative studies have begun to highlight the diversity of individuals aging with HIV, in terms of behavioural factors, social vulnerability, and ethno-racial differences, which might contribute to differences in patterns of aging[1]. Comparative studies within groups of older people with HIV could help us understand relationships between age and other determinants of health, such as treatment history, diagnosis and presentation of comorbidities and, last but not least, duration of HIV disease. Indeed, it has been suggested that people aging with long-term HIV infection and treatment might have characteristically different health and care needs compared with people who have seroconverted at older ages, but evidence in this area is needed[1, 2].

The objective of our study was to assess and compare the prevalence of and risk factors for individual NICM and MM among a group of HIV-positive middle-aged and older adults with a longer duration of HIV infection, and among a group of HIV-positive middle aged and older adults who seroconverted at an older age. We compared estimates across both groups to a matched community-based cohort sampled from the general population.

## METHODS

This study took place within the multidisciplinary Modena HIV Metabolic Clinic (MHMC) cohort study, which was initiated in 2004 to assess longitudinal metabolic changes among people attending the HIV metabolic clinic at the University of Modena and Reggio Emilia School of Medicine. As described elsewhere[11, 15], patients undergo annual multidisciplinary assessments and consultations in multiple domains, including metabolic and endocrinological measures, bone mineral density, organ function, and social factors.

Over the past 10 years an expected shift in age distribution was observed among people attending the MHMC: in 2003 median age was 40 years (interquartile range 37–44) and median age at HIV diagnosis was 33 years; in 2012 median age was 48 years (interquartile range 45–53) and median age at HIV diagnosis was 43 years.

Participants in the MHMC cohort were eligible for inclusion in the current cross-sectional study if their duration of HIV infection at 2013 study visit was within either the 1^st^ or 4^th^ quartile of the cohort. Duration of HIV infection was calculated as the time between HIV diagnosis and 2013 study visit. We then created two groups, matched on age, gender, race (all Caucasian/white), and geographical area of origin: one group of participants who were HIV seropositive for ≥ 20.6 years (an aging with HIV group, “HIV-Aging”), and a second group of participants who were seropositive for < 11.3 years (an aged at HIV seroconversion group, “HIV-Aged”). In consideration of the natural history of untreated HIV disease, and the average length of time between seroconversion and HIV diagnosis, these two statistically driven categories were selected to represent a non-overlapping cohort of participants who acquired HIV in different time periods.

HIV-Aging and HIV-Aged participants were matched in a 1:3 ratio with participants sampled from the general population in the CINECA ARNO database, on age, gender, and race and geographical area of origin[11]. The ARNO Observatory is an on-line, multi-centre observational database in which population-based data is collected and epidemiological methods are used to combine and aggregate large volumes of health and healthcare-related data for each individual participant[16]. These data include primary care provider-generated medication prescriptions, inpatient hospital records and discharge, summaries, diagnostic laboratory tests and radiographic examinations. This information is linked to other sources of participant data (including vital statistics and demographics) in order to provide comprehensive tracking of clinical diagnoses and healthcare use trends throughout Italy. Lifestyle/behavioural, anthropometric and metabolic data are not collected in the CINECA ARNO database.

### Outcomes

NICM diagnoses were based according the following criteria previously used in our studies[11]. The category of CVD included the following diagnoses: myocardial infarction, coronary artery disease, peripheral vascular disease, stroke, angina pectoris, coronary artery bypass grafting, and angioplasty. HTN was defined as blood pressure >140/90 mmHg over two consecutive measurements, T2DM as fasting serum glucose levels >126 mg/dL, and CKD as eGFR<60 ml/min using the MDRD estimating equation. Hypertension and T2DM diagnoses were also identified through current use of antihypertensive and hypoglycemic drugs. In the Aging and Aged HIV-positive groups we also analysed low BMD (t-score<-2SD) using Dual-energy X-Ray Absorptiometry.

### Covariates

Demographic and health variables were characterized and compared between HIV-Aging and HIV-Aged groups at 2013 study visit. These include age, gender, current and nadir CD4 cell counts, plasma HIV RNA viral load, and current smoking habits, as well as anthropometric (body mass index [BMI], waist circumference, lipodystrophy) and cardiovascular and metabolic markers (triglycerides, cholesterol, fasting glucose, and HOMA).

### Statistical analysis

Comparisons between groups were performed using χ^2^ test for categorical variables with Bonferroni adjusted post-hoc analyses (significant level fixed at p<0.017) and T-test or Mann-Whitney U-test for normally and non-normally distributed continuous variables, respectively.

The probability of MM at each age was compared across HIV-Aging, HIV-Aged, and HIV-negative groups using logistic regression models, and univariate and multivariable logistic regression models were constructed to determine odds ratios (ORs) for factors associated with MM risk.

A model was built to compare prevalence of NICM and MM in HIV-Aging and HIV-Aged groups using the HIV-negative group as reference after correction for gender, age (in years), and CD4 cell count <200 cell/μL. Per protocol, we assumed that, in controls, the values for ART exposure and nadir CD4 cell count <200 cells/lL were equal to zero.

A second model, restricted to the HIV-positive groups only, examined the probability of MM taking into account further HIV-related variables including current CD4 lymphocyte cell count/mm3, nadir CD4 cell count, lipodystrophy phenotype, and cumulative exposure to the antiretroviral (ARV) agent drug classes protease inhibitors (PI), nucleoside reverse transcriptase inhibitors (NRTI), non-nucleoside reverse transcriptase inhibitors (NNRTI), fusion inhibitors (FI), or integrase inhibitors (INT). Age was expressed in years; duration of individual ART drug class use was expressed in months; CD4 nadir <200/μL was expressed as a binary variable.

We performed a second subanalysis restricted to elderly HIV-positive patients using the median age as a cut off (45 years) to better estimate the role of aging with HIV, if any.

Statistical analyses were performed STATA Software package, Intercooled version 13.1 for Mac (Stata Corp ltd, Collage Station, TX, USA).

### Ethics

Approval for the Modena HIV Metabolic Clinic cohort study was obtained from the Research Ethics Board of the University of Modena and Reggio Emilia, and all participants provided written consent at their initial clinic visit.

## RESULTS

We analyzed data from 404 HIV-Aging participants and 404 HIV-Aged participants and compared them with data from 2424 control subjects. Per matching criteria, in each group mean age was 46.7±6.2 years, and 28.9% were women.

Table 1 describes demographics and HIV characteristics of the HIV-positive groups.

**Table 1:** Characteristics of HIV-Aging and HIV-Aged groups.

	HIV-Aging	HIV-Aged	p-value
Sample size, n (%)	404	404	—
Men, n (%)	287 (71)	287 (71)	1
Age, mean ± S.D.	47 ± 6	47 ± 6	1
**Current smoke, n (%)**			**0.017**
None	192 (49)	207 (60)	
Moderate*	71 (18)	45 (13)	
Intense *	122 (31)	93 (26)	
**Anthropometry**			
BMI (kg/m^2^) (± S.D.)	23 (± 4)	25 (± 5)	**<0.001**
Waist circumference (cm) (± S.D.)	87 (±10)	89 (±12)	**0.007**
**Lipodistrophy, n (%)**			**<0.001**
No lipodistrophy	46 (12)	83 (23.2)	
Lipoatrophy	160 (41)	101 (28)	
Fat accumulation	180 (46)	173 (48)	
**Cardiovascular and metabolic markers**			
Triglycerides (mmol/L) median (IQR)	150 [105–230]	146 [100–230]	0.83
Total Cholesterol (mmol/L) mean ± S.D.	183 ± 44	199 ± 50	**<0.001**
High Density Lipoprotein (mmol/L), mean ± S.D.	45 ± 14	45 ± 13	0.7
Low Density Lipoprotein (mmol/L), mean ± S.D.	106 ±35	119 ±35	**<0.001**
Fasting Glucose (mmol/L), median (IQR)	91 [85–101]	92 [86–100]	0.96
HOMA, median (IQR)	3.5 [2.1–5.2]	2.6 [1.5–4.2]	**0.001**

* *Moderate smoking is <10 cigarettes per day; intense smoking is ≥10 cigarettes per day*.

Anthropometric and cardio-metabolic characteristics differed between HIV-Aging and HIV-Aged groups. Participants in the HIV-Aging group generally exhibited a higher prevalence of lipoatrophy, lower BMI, and higher rates of insulin resistance than HIV-Aged participants.

**Fig 1:**
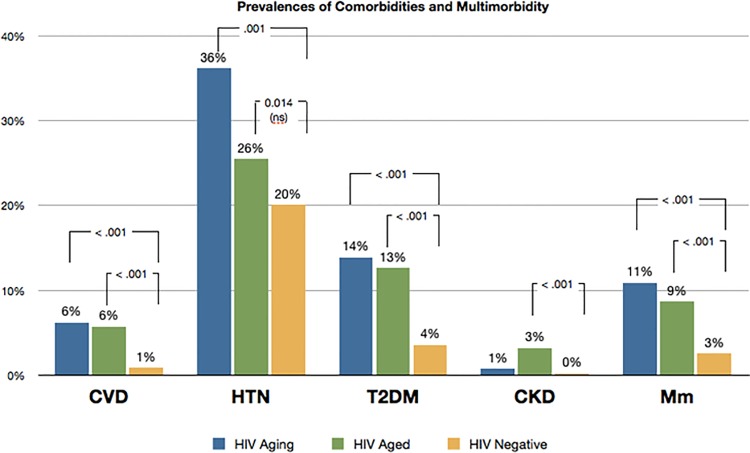
Prevalence of NICM and MM of HIV-Aging, HIV-Aged and HIV-negative groups.

Prevalence of NICM was significantly higher in the HIV-positive groups compared to the HIV-negative group from the general population MM was more common among both HIV-positive groups than the HIV-negative group (p<0.001 for all comparisons; Fig 1).

**Fig 2:**
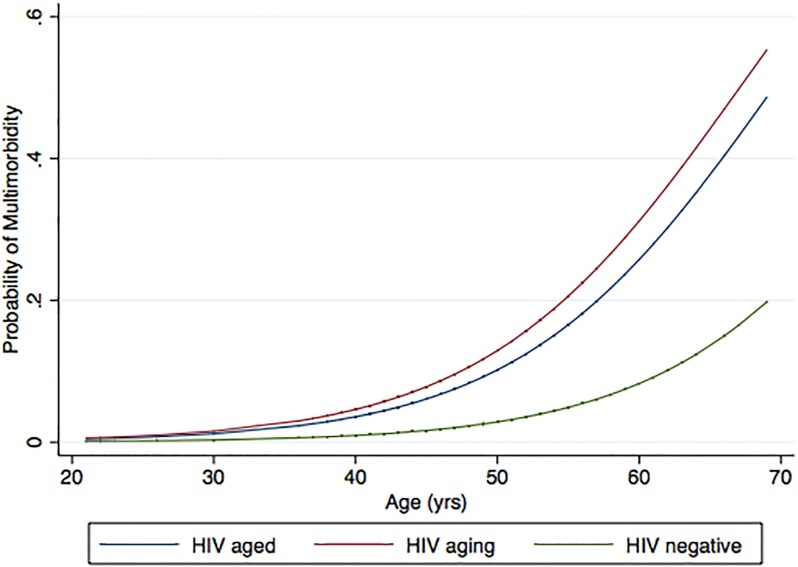
Probability for MM in the three comparative groups across age distribution.

Fig 2 depicts probability of MM in the three comparative groups across age distribution.

At any age the risk for MM was accentuated in HIV-Aging participants compared to HIV-negative individuals, and HIV-Aged participants had an intermediate risk between the other two.

**Fig 3:**
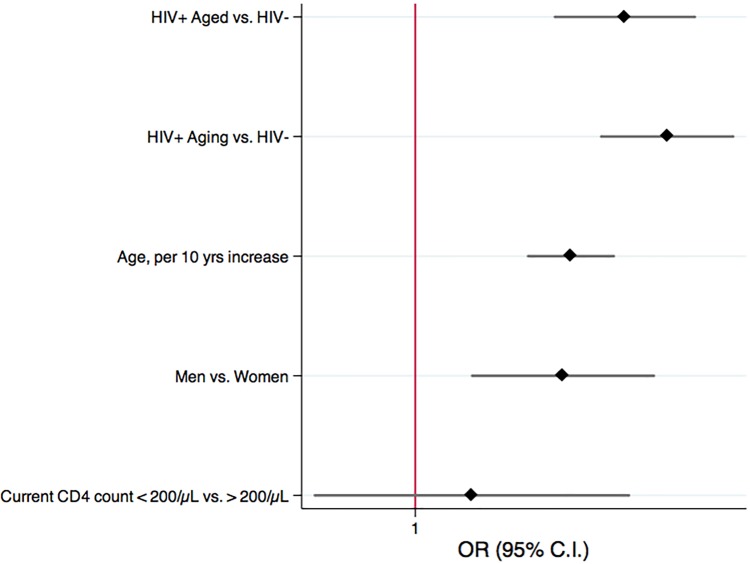
Multivariable logistic regression model to detect independent predictors of MM in the HIV sample.

In multivariable models including age, gender, and nadir CD4 count, we observed 5-fold increased odds of MM in HIV-Aging participants compared to HIV-negative individuals (OR = 5.0, 95% CI,3.3–7.6, p<0.01), and 4-fold increased odds of MM in HIV-Aged participants compared to HIV-negative (OR = 3.8, 95% CI,2.5–6.0, p<0.01) (Fig 3).

Comparing Aging and Aged group, the former had higher rates of hypertension (36.0 vs 26.0, p = 0.001) and a trend towards less prevalence in MM was sown in the latter (10.9 vs 8.7, p = 0.286) (Fig 1).

**Fig 4:**
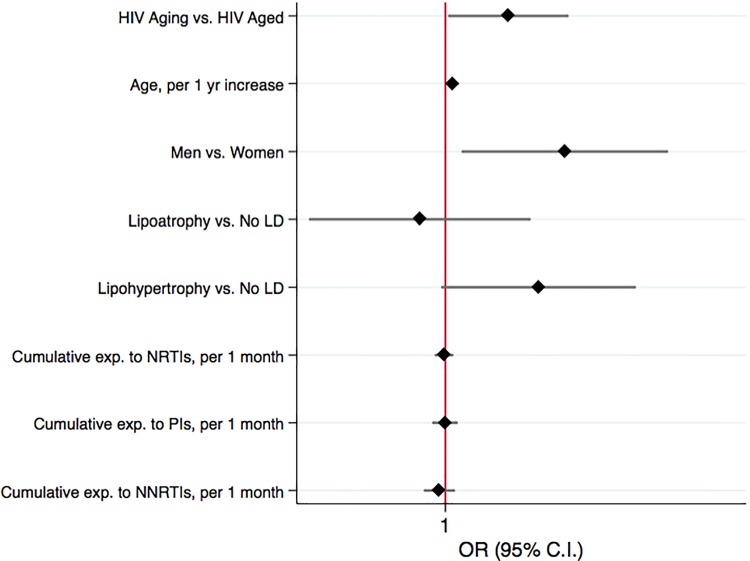
Multivariable logistic regression model to detect independent predictors of MM among HIV-positive participants over 45 years old.

In the subset of HIV patients over-45 years old, including 502 individuals, prevalence of MM in HIV-Aged vs. HIV-Aging was 42% and 58%, (p = 0.181). In multivariable logistic regression we documented a statistically significant increased risk of MMin HIV-Aging compared to HIV-Aged patients (OR 1.92, 95% C.I. 1.03–3.56, p = 0.039) after adjustment for gender (OR 3.46, 95% C.I. 1.19–10.04, p = 0.023), age (per 1 yr increase OR 1.08, 95% C.I. 1.02–1.14, p = 0.004), lipodystrophy, and cumulative exposure to ARVs (Fig 4).

## DISCUSSION

Data from the Modena HIV Metabolic Clinic cohort study show that HIV-infected people are getting older, exhibiting an increased risk for age-associated chronic diseases compared to individuals of the same age sampled from the general population. In particular, this study identified that people with longer duration of HIV infection had relatively higher rates of hypertension and MM than people who seroconverted at older ages.

At any age the risk for individual NICM as well as MM was 5 fold higher in people aging with a longer duration of HIV infection compared to people without HIV, while HIV-positive individuals who seroconverted at an older age had an intermediate risk.

Our results should be interpreted with caution, in particular with regard to reproducibility of our results in different HIV settings. It has been argued that the tertiary referral setting of the MHMC may concentrate patients with higher prevalence of NICM and MM, However as previously demonstrated in a sensitivity analysis this is not the case when comparing the prevalence of these conditions in patients referred from other centres[11]. In Italy, people with HIV have full free access not only to HAART but also to clinical care including diagnostic procedures with no co-pay. This might result in a selection bias as screening activities are offered relatively more frequently to patients with HIV than the general population which might result in higher rates of incidentally identified asymptomatic disease. The information available in the CINECA-ARNO administrative database limited further comparisons in rates of NICM diagnoses, as not all major risk factor for NICM are collected and clinical assessments are not provided. Moreover, with regard to CKD, the number of clinical events were very limited and BMD measurements were not available in CINECA cohort. Finally, the cross-sectional design of our study cannot prove causality regarding the impact of HIV duration on NICM or MM risk, nor we were able to collect biomarkers of systemic and tissue inflammation to argue a pathogenic link between HIV-related factors and MM.

Investigations of disease in people aging with HIV have so far primarily been limited to comparisons between older and young HIV-positive persons, or else between older adults with and without HIV[1]. Studies like the present one which investigate differences among groups of people aging with HIV can yield greater insights into relationships between age, HIV duration, and health, and help identify which patients might be more vulnerable, in whom more preventive interventions could be directed.

As universal access to HIV treatment and care continue to expand, the population of older people with HIV will keep growing. A particular concern in this population regards MM as a predictor care complexity and polypharmacy increasing the cost of clinical management[17]. Moreover polifarmacy rises risks of pharmacological interactions and medical errors[18–20]. Ageing and chronic HIV infection can also both contribute to subclinical changes, including atherosclerosis, bone mineral density, and changes in T cell function.

Very few studies examined the impact of clinical intervention on both physical and neurocognitive impairment associated with aging[21–25].

Our results help reinforce the idea that as a group, people aging with HIV remain heterogeneous in terms of health. Factors such as longer duration of HIV treatment might be used in clinical practice to inform clinical surveillance of disease risk, suggests frequency of follow-up, intensity of care and treatment.

In conclusion, we identified that older HIV-positive adults exhibit higher rates of age-associated chronic conditions and MM than HIV-negative individuals of the same age, further, that people with longer duration of HIV infection show the higher probability of MM than people who seroconverted at older ages.

We recommend that future longitudinal studies on older age and HIV reflect holistic views of health, investigate the diversity of people aging with HIV, and take particular consideration of the more vulnerable subset of people aging with longer duration of HIV infection.

## References

[1] Chambers LA, Wilson MG, Rueda S, Gogolishvili D, Shi MQ, Rourke SB, et al Evidence informing the intersection of HIV, aging and health: a scoping review. AIDS and behavior. 2014;18(4):661–75. 10.1007/s10461-013-0627-5 . 24185708

[2] Lazarus JV, Nielsen KK. HIV and people over 50 years old in Europe. HIV medicine. 2010;11(7):479–81. 10.1111/j.1468-1293.2009.00810.x . 20136658

[3] May M, Gompels M, Delpech V, Porter K, Post F, Johnson M, et al Impact of late diagnosis and treatment on life expectancy in people with HIV-1: UK Collaborative HIV Cohort (UK CHIC) Study. Bmj. 2011;343:d6016 10.1136/bmj.d6016 21990260 PMC3191202

[4] Guaraldi G, Cossarizza A, Franceschi C, Roverato A, Vaccher E, Tambussi G, et al Life expectancy in the immune recovery era: the evolving scenario of the HIV epidemic in northern Italy. Journal of acquired immune deficiency syndromes. 2014;65(2):175–81. 10.1097/QAI.0000000000000018 . 24442223

[5] Mills EJ, Bakanda C, Birungi J, Chan K, Ford N, Cooper CL, et al Life expectancy of persons receiving combination antiretroviral therapy in low-income countries: a cohort analysis from Uganda. Annals of internal medicine. 2011;155(4):209–16. 10.7326/0003-4819-155-4-201108160-00358 . 21768555

[6] Mutevedzi PC, Newell ML. A missing piece in the puzzle: HIV in mature adults in sub-Saharan Africa. Future virology. 2011;6(6):755–67. 10.2217/fvl.11.43 22427781 PMC3303125

[7] Cooperman NA, Arnsten JH, Klein RS. Current sexual activity and risky sexual behavior in older men with or at risk for HIV infection. AIDS education and prevention: official publication of the International Society for AIDS Education. 2007;19(4):321–33. 10.1521/aeap.2007.19.4.321 17685845 PMC2505189

[8] CDC, Center for Disease Control and Prevention (2008) HIV/ AIDS among Persons Aged 50 and Older.

[9] Mahy M, Autenrieth CS, Stanecki K, Wynd S. Increasing trends in HIV prevalence among people aged 50 years and older: evidence from estimates and survey data. Aids. 2014;28 Suppl 4:S453–9. 10.1097/QAD.0000000000000479 25222641 PMC4247270

[10] Onen NF, Overton ET, Seyfried W, Stumm ER, Snell M, Mondy K, et al Aging and HIV infection: a comparison between older HIV-infected persons and the general population. HIV clinical trials. 2010;11(2):100–9. 10.1310/hct1102-100 . 20542846

[11] Guaraldi G, Orlando G, Zona S, Menozzi M, Carli F, Garlassi E, et al Premature age-related comorbidities among HIV-infected persons compared with the general population. Clinical infectious diseases: an official publication of the Infectious Diseases Society of America. 2011;53(11):1120–6. 10.1093/cid/cir627 . 21998278

[12] Schouten J, Wit FW, Stolte IG, Kootstra NA, van der Valk M, Geerlings SE, et al Cross-sectional comparison of the prevalence of age-associated comorbidities and their risk factors between HIV-infected and uninfected individuals: the AGEhIV cohort study. Clinical infectious diseases: an official publication of the Infectious Diseases Society of America. 2014;59(12):1787–97. 10.1093/cid/ciu701 . 25182245

[13] Althoff KN, Jacobson LP, Cranston RD, Detels R, Phair JP, Li X, et al Age, comorbidities, and AIDS predict a frailty phenotype in men who have sex with men. The journals of gerontology Series A, Biological sciences and medical sciences. 2014;69(2):189–98. 10.1093/gerona/glt148 24127428 PMC4038242

[14] Samji H, Cescon A, Hogg RS, Modur SP, Althoff KN, Buchacz K, et al Closing the gap: increases in life expectancy among treated HIV-positive individuals in the United States and Canada. PloS one. 2013;8(12):e81355 10.1371/journal.pone.0081355 24367482 PMC3867319

[15] Guaraldi G, Orlando G, Squillace N, De Santis G, Pedone A, Spaggiari A, et al Multidisciplinary approach to the treatment of metabolic and morphologic alterations of HIV-related lipodystrophy. HIV clinical trials. 2006;7(3):97–106. 10.1310/EYWJ-8B5K-X7VQ-9CPE . 16880166

[16] CINECA ARNO Observational database. Available at: https://osservatorioarno.cineca.org. Accessed: 09 August 2014

[17] Guaraldi G, Zona S, Menozzi M, Carli F, Bagni P, Berti A, et al Cost of noninfectious comorbidities in patients with HIV. ClinicoEconomics and outcomes research: CEOR. 2013;5:481–8. 10.2147/CEOR.S40607 24098086 PMC3789842

[18] Krentz HB, Gill MJ . Increased costs of HIV care associated with aging in an HIV-infected population. HIV medicine. 2015;16(1):38–47. 10.1111/hiv.12176 . 25105798

[19] Greene M, Justice AC, Lampiris HW, Valcour V. Management of human immunodeficiency virus infection in advanced age. Jama. 2013;309(13):1397–405. 10.1001/jama.2013.2963 23549585 PMC3684249

[20] Levett T WJ, Fisher M. HIV and ageing: what the geriatrician needs to know. Rev Clin Gerontol 2014 24:10–24.

[21] Bhaskaran K, Mussini C, Antinori A, Walker AS, Dorrucci M, Sabin C, et al Changes in the incidence and predictors of human immunodeficiency virus-associated dementia in the era of highly active antiretroviral therapy. Annals of neurology. 2008;63(2):213–21. 10.1002/ana.21225 . 17894380

[22] Heckman TG, Sikkema KJ, Hansen N, Kochman A, Heh V, Neufeld S, et al A randomized clinical trial of a coping improvement group intervention for HIV-infected older adults. Journal of behavioral medicine. 2011;34(2):102–11. 10.1007/s10865-010-9292-6 20857188 PMC3247911

[23] Heckman TG, Barcikowski R, Ogles B, Suhr J, Carlson B, Holroyd K, et al A telephone-delivered coping improvement group intervention for middle-aged and older adults living with HIV/AIDS. Annals of behavioral medicine: a publication of the Society of Behavioral Medicine. 2006;32(1):27–38. 10.1207/s15324796abm3201_4 . 16827627

[24] Heckman TG, Kochman A, Sikkema KJ, Kalichman SC, Masten J, Bergholte J, et al A pilot coping improvement intervention for late middle-aged and older adults living with HIV/AIDS in the USA. AIDS care. 2001;13(1):129–39. 10.1080/09540120020018233 . 11177470

[25] Neundorfer MM, Camp CJ, Lee MM, Skrajner MJ, Malone ML, Carr JR. Compensating for cognitive deficits in persons aged 50 and over with HIV/AIDS: a pilot study of a cognitive intervention. Journal of HIV/AIDS & Social Services. 2004;3(1):79–97.

